# Combining the Telomerase Inhibitor Imetelstat with the BCL-2 Inhibitor Venetoclax Enhances Apoptosis and Improves Survival in Acute Myeloid Leukemia Cellular and Animal Models

**DOI:** 10.3390/jcm15197399

**Published:** 2026-09-23

**Authors:** Fei Huang, Joshua J. Rusbuldt, Leopoldo Luistro, Heidi Colton, Joseph Eid

**Affiliations:** 1 Geron Corporation, Foster City, CA 94404, USA; fay_f_huang@hotmail.com (F.H.); jeid@geron.com (J.E.); 2 Janssen Research & Development, LLC, Spring House, PA 19477, USA

**Keywords:** acute myeloid leukemia, apoptosis, BCL-2, imetelstat, mechanism of action, synergy, telomerase, venetoclax

## Abstract

**Background/Objectives**: Acute myeloid leukemia (AML) is a heterogeneous disease with limited treatment options. New therapies with novel mechanisms of action are needed. Telomerase and BCL-2 are overexpressed in AML and correlate with disease severity and poor prognosis, suggesting dual targeting may be beneficial. **Methods**: We evaluated the effect of imetelstat, a telomerase inhibitor, in combination with venetoclax, a BCL-2 inhibitor, in AML cell lines, AML patient-derived samples, and in a MOLM-13 AML xenograft model. **Results**: In AML cell lines and patient samples, imetelstat plus venetoclax demonstrated dose-dependent synergistic activity to induce apoptosis. Studying AML patient samples ex vivo showed the effect is preferentially in CD34^+^ leukemic cells, and not in lymphocytes. The synergy induced by venetoclax plus imetelstat was stronger than venetoclax plus hypomethylating agents in AML cell lines. The in vivo MOLM-13 AML xenograft model demonstrated that imetelstat plus venetoclax prolonged survival of tumor-bearing mice compared to either agent alone. Forty percent of mice in the combination group were still alive eighty days after the end of treatment, whereas all mice in the single-agent groups succumbed to disease within two weeks. Mechanisms of synergy elicited by the combination were further illustrated through reduction of hTERT expression and telomerase activity, decreases in the interaction of BCL-2 with BAX, and enhanced mitochondrial dysfunction via loss of mitochondrial membrane potential, and these led to marked increases in the intrinsic mitochondrial apoptosis pathway in AML cells. **Conclusions**: This study supports the clinical evaluation of imetelstat plus venetoclax for the treatment of AML in the Phase 1 IMAGINE trial (NCT07320235).

## 1. Introduction

Acute myeloid leukemia (AML) is an aggressive, heterogeneous disease, historically with limited treatment options [1,2]. Despite the recent approval of a number of targeted therapies for defined molecular subtypes, an unmet need still exists for new agents with novel mechanisms of action and combination regimens that maximize efficacy while preserving tolerability [3,4].

Expression of telomerase is high in AML, as in most tumors, but is almost undetectable in the majority of normal somatic cells, making it an attractive cancer target [5,6,7]. Telomerase is an enzyme complex comprised of a variety of molecular players, most notably the enzyme component itself, hTERT, and the RNA template, hTR or TERC [5]. This enzyme complex binds to the terminal ends of chromosomes (telomeres) via complementary base pairing to the hTR nestled in the active pocket of hTERT and iteratively incorporates the hexamer TTAGGG string of nucleotides to elongate and maintain telomere length [5]. In addition to this canonical function of telomere elongation, hTERT/telomerase has many other biological activities. For example, hTERT has been observed to enhance survival, chemoresistance, invasion, and metastasis of malignant cells independent of its telomere lengthening function [8,9,10]. Furthermore, hTERT can translocate to the mitochondria during oxidative stress, reducing reactive oxygen species (ROS) levels and protecting mitochondrial DNA, which helps cancer cells survive metabolic stress [11,12].

Imetelstat is a novel, first-in-class, competitive inhibitor of telomerase activity. Structurally, imetelstat is a lipidated 13-mer oligonucleotide complementary to the hTR template that inhibits the interaction of the active site with telomere ends [13]. Imetelstat has demonstrated broad activity in vitro and in vivo against a variety of tumor types, including AML [13,14,15]. Imetelstat further demonstrated clinical benefits in myeloproliferative neoplasms, particularly essential thrombocythemia, myelofibrosis, and lower-risk myelodysplastic syndrome [16,17,18,19,20,21]. Imetelstat is currently approved for the treatment of adult patients with low- to intermediate-1-risk myelodysplastic syndromes with transfusion-dependent anemia (defined in the United States prescribing information as requiring ≥4 red blood cell units over 8 weeks) who are relapsed/refractory to or ineligible for erythropoiesis-stimulating agents [22,23]. Approval was based on the Phase 3 IMerge trial (NCT02598661) that demonstrated greater ≥8-week, ≥24-week, and ≥1-year (primary, secondary, and exploratory end points, respectively) red blood cell transfusion independence rates versus placebo and a low rate of progression to AML (2% in both groups) [18].

BCL-2 is an antiapoptotic protein that promotes the survival of AML cells, particularly leukemic stem cells, by inhibiting apoptosis, and its overexpression is associated with drug resistance and poor prognosis in AML [24]. Venetoclax is a BH3 mimetic small molecule drug that binds and antagonizes BCL-2 to induce cell death via the intrinsic apoptosis pathway [25]. Preclinical studies have shown a differential response of AML tumor cell lines and primary patient samples to venetoclax [26], and clinical studies indicated modest single-agent activity, particularly in relapsed/refractory cases [24,27,28]. Currently, venetoclax in combination with azacitidine is a standard-of-care regimen for newly diagnosed patients ≥ 75 years of age with AML who are not eligible for intensive chemotherapy based on the results of the Phase 3 VIALE-A study (NCT02993523), with venetoclax approved for use with either azacitidine or decitabine [29,30,31,32]. In this trial, significantly higher composite complete remission (defined as complete remission or complete remission with incomplete hematologic recovery) rates, along with longer median overall survival, were achieved with venetoclax plus azacitidine compared with placebo plus azacitidine [29,31].

The telomerase component hTERT contains a BH3-like motif that allows it to directly interact with antiapoptotic proteins, such as BCL-xL and MCL-1, and this interaction acts to suppress the mitochondrial apoptosis pathway [33,34,35]. hTERT has been found in the mitochondria, where it acts to protect against stress-induced apoptosis by inhibiting the release of proapoptotic factors, often leading to increased levels of BCL-2 and decreased BAX/BCL-2 ratios [34]. Preclinical evidence showed that downregulation of hTERT induces apoptosis via disruptions of hTERT and interactions with BCL-2 [36]. Studies have also shown that overexpression of BCL-2 in cancer cells can lead to enhanced telomerase activity, overexpression of the *hTERT* gene, and increased hTERT protein; conversely, lowering BCL-2 levels can reduce telomerase activity [35,37], all suggesting cross-talk between hTERT and BCL-2. This led us to hypothesize that inhibiting both targets would yield greater antitumor activity in AML. To this end, we performed preclinical studies to investigate if combining imetelstat with venetoclax could enhance induction of apoptosis in AML cell lines in vitro and in patient samples ex vivo and if this combination could prolong survival of xenograft models in vivo. We further investigated the molecular mechanisms underlying possible synergistic activity of imetelstat plus venetoclax compared with venetoclax plus hypomethylating agents in AML cell lines.

## 2. Materials and Methods

### 2.1. AML Patient Samples

Peripheral blood mononuclear cells (PBMCs) from four patients with AML were purchased from a commercial supplier (Discovery Life Sciences, Huntsville, AL, USA). Discovery Life Sciences obtained the biospecimens under Institutional Review Board (IRB)/Ethics Committee oversight and obtained informed consent from donors. Patient and disease characteristics from de-identified donors, as provided by the commercial supplier, are listed in the Supplementary Methods.

### 2.2. In Vitro and Ex Vivo Drug Treatment

AML cell lines were obtained from American Type Culture Collection (Manassas, VA, USA) or Deutsche Sammlung von Mikroorganismen und Zellkulturen (Braunschweig, Germany) and grown in their respective culture media in 96-well culture plates at 20,000 cells per well at 37 °C and 5% CO_2_. Mycoplasma negativity was confirmed after thawing all parent cultures. PBMCs were purified from the blood of patients with AML by Ficoll gradient and seeded in RPMI-1640 supplemented with 10% fetal bovine serum at 300,000 cells per well. Powdered imetelstat, mismatch oligonucleotide (where 5 bases in the sequence are swapped), and sense oligonucleotide (where the sequence is reversed) controls were sourced by Janssen and dissolved in nuclease-free, sterile water. Venetoclax (Selleckchem, Houston, TX, USA) was dissolved in dimethyl sulfoxide. Drugs in different dilutions were added to cell cultures either as single agents or in combination after seeding, and plates were incubated at 37 °C for different durations, as indicated in figure legends, before each individual end point was assessed. Drug combination effects using the full dose range of each drug for potential synergy were analyzed utilizing the web-based Horizon CombinatoRx with Chalice^TM^ analyzer application (Horizon Discovery Inc., Cambridge, UK) that performs isobolar analysis based on methods described previously [38].

### 2.3. Apoptosis Assay

Cell lines or PBMCs from patients with AML after drug treatment were assayed for cell viability using flow cytometric assessment of Annexin V and propidium iodide (BioLegend, San Diego, CA, USA) on a BD FACSCanto II flow cytometry system (BD Biosciences, Franklin Lakes, NJ, USA), as kit protocols describe.

### 2.4. In Vivo Study in AML Xenograft Models

All animal work was performed in accordance with the Guide for the Care and Use of Laboratory Animals and approved by the Institutional Animal Care and Use Committee of Janssen R&D in Spring House, Pennsylvania, and animals were housed and cared for in species-appropriate conditions, as per these guidelines. Female SCID-beige mice (Charles River Laboratories, Wilmington, MA, USA) were treated at approximately 6 to 8 weeks of age (20–22 g body weight). Mice were injected with one million MOLM-13 cells intravenously via the lateral tail vein on day 0, and 10 mice were randomly assigned to each group. The drug treatment started on day 1, with mice dosed with freshly prepared single agents, the combination, or vehicle. Imetelstat and mismatch controls were administered intraperitoneally at 30 mg/kg body weight three times weekly for four weeks, and venetoclax and vehicle (i.e., 60% Phosal50, 30% polyethyleneglycol-400, and 10% ethanol) were administered daily at 100 mg/kg via oral gavage for 4 weeks. Animals were monitored twice weekly for body weight and daily for adverse clinical effects of tumor burden, and animals losing greater than 20% of their body weight were removed from the study. Increased life span (ILS) analysis was performed as described [39] and log-rank (Mantel–Cox) tests were used to determine *p*-values. Statistical analyses were performed using GraphPad Prism, version 7.0.

### 2.5. Assay for Telomerase Activity

Cell pellets were lysed in a modified NP-40 buffer, as described previously [40], and quantified for protein using a reducing-agent-compatible BCA assay (Thermo Fisher Scientific, Waltham, MA, USA). Relative telomerase activity was measured via qPCR-TRAP using primers previously reported [40] with Power SYBR Green Master Mix (Thermo Fisher Scientific) based on the manufacturer’s protocols on the ViiA7 Real-Time PCR system (Thermo Fisher Scientific). Relative telomerase activity was determined by comparing tested conditions against a standard curve derived from control lysates (HeLa derived), and these were normalized as percentages compared to untreated controls. Relative telomerase activity was defined as described previously [41]:

$$10^{\frac{\mathrm{Mean}{\mathrm{Ct}}_{\mathrm{Sample}}-y{\mathrm{intercept}}_{\mathrm{Standard}\mathrm{Curve}}}{{\mathrm{Slope}}_{\mathrm{Standard}\mathrm{Curve}}}}.$$

### 2.6. Reverse Transcription–Quantitative Polymerase Chain Reaction (RT-qPCR) Assays for Expression of hTERT and Bcl-2 Family Genes

Cells were grown in flasks and subjected to single-agent or combination treatment for the times indicated in the figure legends. RNA was isolated using the AllPrep Mini Kit (Qiagen, Hilden, Germany; #80204), as the kit protocol described, and gene expression was assessed by RT-qPCR using the High-Capacity cDNA Reverse Transcription Kit (Thermo Fisher Scientific), as the kit protocol described, using the ViiA7 Real-Time PCR system. TaqMan kits were from Thermo Fisher Scientific for BCL-2 (Assay ID Hs00608023_m1), *MCL1* (Assay ID Hs00172036_m1), *BCL2L1*/*BCL-xL* (Assay ID Hs00236329_m1), *BCL2A1*/*Bfl1* (Assay ID Hs00187845_m1), and glyceraldehyde 3-phosphate dehydrogenase (GAPDH; Assay ID Hs01922876_u1). For hTERT, RNA was reverse-transcribed, and cDNA was preamplified using the TaqMan PreAmp Master Mix Kit (Thermo Fisher Scientific), as the kit protocol described. Preamplified cDNA was subjected to TaqMan expression analysis using primers and probes previously described [42] on the ViiA7 Real-Time PCR system with GAPDH as a housekeeping control. Changes in expression were determined using the ΔΔCt method [43].

### 2.7. Flow Cytometry to Assess BAX Protein Expression and Activation

MOLM-13 cells were preincubated with caspase inhibitor Z-VAD-FMK (Promega, Madison, WI, USA) for 2 h followed by single-agent or combination treatment overnight. Cells were analyzed by flow cytometry on the FACs Celesta system (BD Biosciences) after live/dead staining (Thermo Fisher Scientific) for 10 min, fixation and permeabilization for 30 min, and staining for total BAX (BioLegend) or conformationally active BAX (Trevigen, Gaithersburg, MD, USA) for 30 min [44]. Data were analyzed in Cytobank (Beckman Coulter Life Sciences, Indianapolis, IN, USA).

### 2.8. Fluorescent Microscopy to Detect Protein Expression in Cellular Locations

BAX and hTERT expression and cell localization were assessed by fluorescent microscopy. Briefly, cells were stained with MitoTracker^TM^ Deep Red FM (Thermo Fisher Scientific), then fixed with 3% paraformaldehyde in phosphate-buffered saline (PBS) for 15 min. After quenching with ammonium chloride, cells were permeabilized with 0.25% gelatin and 0.3% saponin in PBS for 20 min. Primary antibodies against hTERT (Rockland Immunochemicals, Pottstown, PA, USA) and BAX (Abcam, Cambridge, UK) were incubated in permeabilization buffer for 1 h then probed with secondary antibodies, anti-rabbit AF488 (Thermo Fisher Scientific) or anti-mouse AF594 (Thermo Fisher Scientific), for 30 min with Hoechst 33342 co-staining (Thermo Fisher Scientific). Cells were imaged with the OperaLX (PerkinElmer, Shelton, CT, USA) fluorescent microscope (40× objective), and images were false colored and overlaid with PerkinElmer’s Columbus software v2.91.

### 2.9. Assays to Detect Mitochondrial Membrane Potential

MOLM-13 cells were treated with the single agents or combination for up to 48 h, followed by 30 min of 2 μM JC-1 from the MitoProbe Assay Kit (Thermo Fisher Scientific). Cells were washed free of drug and dye before staining with Annexin V-APC (BioLegend) for 15 min and analyzed by flow cytometry on the FACs Celesta analyzer and interpreted as described [36].

### 2.10. Western Blotting

MOLM-13 cells were treated for up to 48 h and lysed with RIPA buffer containing HALT protease and phosphatase inhibitors for 15 min on ice and centrifuged. Lysates were resolved by sodium dodecyl sulfate–polyacrylamide gel electrophoresis (SDS-PAGE) on 4% to 12% bis-tris acrylamide gels (Thermo Fisher Scientific), transferred to 0.45 μm nitrocellulose membranes (Thermo Fisher Scientific), and blocked with Odyssey^TM^ PBS Blocking Buffer (LI-COR, Lincoln, NE, USA). Blots were probed for the following: GAPDH (Abcam #ab8245 or Cell Signaling Technology, Danvers, MA, USA #2118S), BAX (BD Biosciences #556467), p53 (Cell Signaling #2524S), Caspase 3 (Cell Signaling Technology #9668S), and γ-H2AX (Cell Signaling Technology #2577S). Primary antibody incubation was performed overnight, and respective secondary antibodies were applied for 1 h: anti-rabbit 800 (LI-COR), anti-mouse 680 (LI-COR), anti-mouse 800 (LI-COR), and anti-rabbit 680 (LI-COR). The relative amounts of proteins were quantified by scanning the blots with the Odyssey gel densitometer scanner (LI-COR) and signal normalization to GAPDH.

### 2.11. Coimmunoprecipitation to Detect BCL-2 and BAX Interaction

MOLM-13 cells were treated for 6 or 24 h, and cell pellets were collected for nondenaturing protein lysis. Lysates were parsed to equal aliquots (approximately 500 μg each) and incubated overnight with BCL-2 (Cell Signaling Technologies #4223S) as capture antibodies. Protein A capture beads (Abcam) were prepared as the kit protocol described and incubated with antibody:lysate mixtures for 1 h, then purified by centrifugation. Capture:prey complexes were eluted by boiling purified beads in 4× NuPAGE LDS Sample Buffer (Thermo Fisher Scientific) and 10× NuPAGE Sample Reducing Agent (Thermo Fisher Scientific) for 5 min. Eluates were resolved by SDS-PAGE, as described above. Blots were probed with anti-BAX antibodies (BioLegend) overnight, and secondary anti-mouse 800 antibodies (LI-COR) were probed for 1 h. Blots were imaged and analyzed as above.

## 3. Results

### 3.1. Combining Imetelstat with Venetoclax Exhibits Enhanced Apoptosis in a Subset of AML Cell Lines

Eight AML lines were screened for sensitivity to imetelstat, venetoclax, or both by measuring viable cells via staining for the apoptotic markers Annexin V and propidium iodide after 96 h of treatment. The timeframe of 96 h was selected to ensure that the treatments at the different doses investigated had sufficient time to induce apoptosis in each of the distinct cell lines tested [45]. Three distinct patterns of susceptibility were observed with the different treatments, as seen in Figure 1, with 0 μM imetelstat representing the dose–response curve for venetoclax alone. Three cell lines (HEL, U937, and NB-4) showed resistance to venetoclax at the dose range tested (Figure 1A) but demonstrated a dose-dependent response (as shown on the *Y* axis) to imetelstat and no apparent added effect of the combination. Three other cell lines (MOLM-13, HL-60, and KG-1) were sensitive to venetoclax and moderately sensitive to imetelstat (except HL-60), but combining both drugs further reduced the percentage of viable cells even in the imetelstat-insensitive HL-60 cell line (Figure 1B). In the third group, two cell lines (OCI-AML3 and OCI-AML5) were resistant to both venetoclax or imetelstat as single agents but showed some reduction in viability with combination treatment, particularly at higher doses of venetoclax (Figure 1C). Half maximal inhibitory concentration (IC_50_) values for the susceptible cell lines (Figure 1B) were 33.5 nM for MOLM-13, 498.2 nM for HL-60, and 89.6 nM for KG-1. IC_50_ values could not be derived for the cell lines in Figure 1A (Group 1) nor Figure 1C (Group 3) due to the insensitivity of the cell lines to venetoclax alone.

The reduced cell viability trended with the enhanced DNA damage, as measured by the increased level of γ-H2AX (Figure 2A). Imetelstat alone had limited effects on all three cell lines, but imetelstat plus venetoclax had a more enhanced effect in MOLM-13 than in OCI-AML3 cells and a less additive effect in HEL cells compared with venetoclax alone. These patterns were similar to the response patterns of reduced cell viability by the combination seen in Figure 1. MOLM-13 cells were more responsive to the combination than OCI-AML3 cells, whereas no added effect was seen in HEL cells. The most prominent increase in γ-H2AX in MOLM-13 cells was also associated with an increase in p53 expression (Figure 2B).

### 3.2. Imetelstat Plus Venetoclax Synergistically Induced Apoptosis in AML Cell Lines

A full dose range combination study in MOLM-13 and HL-60 cell lines was conducted to evaluate whether the combination effect is additive or synergistic. Loewe’s additivity model was utilized to derive synergy scores utilizing Horizon’s Chalice analyzer. If the score is greater than 1, a synergistic interaction between the two drugs is suggested. Consistent with results in Figure 1B, imetelstat plus venetoclax was more active in MOLM-13 than in HL-60 cells (Figure 3A). The sensitivities were dramatically shifted when combining both drugs with lower concentrations than the single agents alone. The calculated synergy scores were 5.12 and 11.33 when MOLM-13 cells were treated with imetelstat plus venetoclax for 48 h and 96 h, respectively (Supplementary Table S1), indicating strong synergistic effects of this combination. By comparison, synergy scores were 0.22 and 0.03 for the mismatch oligonucleotide control and 0.41 and 0.06 for the sense oligonucleotide control in combination with venetoclax for 48 and 96 h of treatment, respectively, indicating imetelstat’s synergistic induction of apoptosis when combined with venetoclax.

Because venetoclax in combination with azacytidine or decitabine was approved for the treatment of patients with newly diagnosed AML based on studies that showed this combination was effective and well tolerated in elderly patients with AML [29,30], we compared the synergistic effects of venetoclax plus imetelstat to the effects of venetoclax plus either azacytidine or decitabine. When MOLM-13 cells were treated with venetoclax and azacytidine or with decitabine for 96 h, the synergy scores were 1.43 and 3.46, respectively (Supplementary Table S2), suggesting less prominent synergistic effects, as compared with the score of 11.33 for venetoclax in combination with imetelstat (Supplementary Table S1). These results were consistent with the combination results in the HL-60 cell line: the synergy scores were 1.44, 2.64, and 3.06 for venetoclax in combination with azacytidine, decitabine, or imetelstat, respectively, for 96 h of treatment (Supplementary Table S3), indicating the synergistic effect is stronger for venetoclax with imetelstat than with the hypomethylation agents in the two AML cell lines tested.

Venetoclax alone induced both Annexin V^+^/propidium iodide^+^ and Annexin V^+^/propidium iodide^−^ populations (Figure 3B), the latter indicating early apoptosis, as per protocol designation (bottom panel), whereas imetelstat alone did not impact this population (left panels; from bottom up is the increased concentration of imetelstat). However, with the addition of imetelstat to venetoclax, this population shifted to double-positive apoptotic cells in a dose-dependent manner, suggesting that imetelstat may alter the threshold of apoptotic induction in these cells. By comparison, azacytidine (Figure 3B, right panels) or decitabine alone only induced early apoptotic cells, which were further enhanced when combined with venetoclax, but conferred no added impact on the double-positive apoptotic cells compared with venetoclax alone.

### 3.3. Imetelstat Plus Venetoclax Synergistically Induced Apoptosis in Primary AML Blasts Ex Vivo

Purified PBMCs from four patients with AML were treated with imetelstat plus venetoclax for 16 or 40 h, were stained with CD45 and CD34, and were assessed for viability by Annexin V/propidium iodide. The survival of SSC^low^/CD45^dim^/CD34^+^ leukemic cells was minimally impacted by imetelstat alone after 16 h of treatment but trended toward a dose-dependent decrease after 40 h—venetoclax showed dose- and time-dependent decreases in survival (Figure 4A). When combining both drugs, a strong reduction in viability was observed, with the highest concentration pair yielding means ± SDs of 26% ± 13% and 6% ± 6% survival at 16 and 40 h, respectively (compared with 50% and 26%, respectively, by venetoclax alone). These results demonstrate that the combination of imetelstat with venetoclax enhanced apoptotic activity in AML patient samples more than either single agent alone, despite variations in response between patients, whereas it had no effect on normal lymphocytes, which is not unexpected, as lymphocytes do not rely on BCL-2 to survive and telomerase is minimally active in mature resting lymphocytes [46,47] (SSC^low^/CD45^hi^; Figure 4B).

### 3.4. Imetelstat in Combination with Venetoclax Prolonged Survival of MOLM-13 AML Xenografts In Vivo

The combined effect of imetelstat and venetoclax was further investigated in vivo in SCID-beige mice injected with MOLM-13 cells. Mice were randomized into 5 groups (10 mice per group) and treated with vehicle control, imetelstat, venetoclax, or a combination of venetoclax with either imetelstat or mismatch oligonucleotide control. Mouse body weights remained relatively consistent for each group for the duration they were monitored (Supplementary Figure S1A), and there was no drug-related toxicity observed. Mice in the vehicle group began to exhibit hind-limb paralysis by day 20, and all died by day 23 due to tumor burden (Figure 4C, left). Mice in the single-agent groups survived longer than those in the vehicle control group, whereas mice in the imetelstat and venetoclax combination group showed prolonged median survival, with 4 out of 10 mice (40%) still alive at the end of the study, which was more than 11 weeks after the last treatment dose (Supplementary Figure S1B). ILS analysis was performed based on criteria set by the National Cancer Institute [39]. Compared with vehicle control, both imetelstat and venetoclax as single agents extended median survival, whereas the combination further enhanced ILS to 68.1% (Figure 4C, right). Furthermore, the combination increased median survival, which translated to 39.6% ILS or 23.3% ILS compared with single-agent imetelstat or venetoclax, respectively.

### 3.5. hTERT Expression and Telomerase Activity Were Reduced with the Combination of Imetelstat and Venetoclax

Imetelstat reduces telomerase activity and decreases hTERT RNA expression levels [14,19,48], so to assess whether venetoclax could enhance these effects, MOLM-13 cells were treated with either imetelstat, venetoclax, or both, and hTERT RNA expression and telomerase activity were measured. Imetelstat alone reduced hTERT RNA expression and telomerase activity at 48 h, with further decreases at 96 h, as expected. Venetoclax alone resulted in a moderate reduction of hTERT and telomerase activity after 96 h of treatment. Combining both drugs dramatically modulated both hTERT and telomerase activity to levels barely detectable by the assays (Figure 5A).

### 3.6. Imetelstat in Combination with Venetoclax Enhanced the Activation of BAX and Reduced the BAX and BCL-2 Interaction

To investigate the effects of imetelstat on apoptosis sensitization, we tested the impact of imetelstat on expression of Bcl-2 family genes and observed a moderate decrease of MCL1, BCL-2, BCL-xL, and BFL1 RNA levels by imetelstat alone or in combination with venetoclax (Supplementary Figure S2A), which translated to appreciable changes at the protein level (Supplementary Figure S2B). Because BAX activation is a critical step in mitochondrial apoptosis [49], we tested if imetelstat could enhance venetoclax-induced BAX activation, as assessed by BAX conformational change. Venetoclax treatment strongly increased the ratio of activated BAX to total BAX in a dose-dependent manner, and imetelstat further enhanced BAX activation, although it did not have an effect as a single agent alone (Figure 5B).

The interaction between prosurvival BCL-2 and apoptotic activator BAX was evaluated via coimmunoprecipitation. Results indicated that the BCL-2 and BAX interaction was reduced by imetelstat or venetoclax as single agents and was further augmented by the combination to roughly 30% of that observed in untreated samples (Figure 5C). Capture of BAX or BAK with apoptotic stimulators, such as NOXA or PUMA, was also performed, but no changes were observed with treatment.

**Figure 5:**
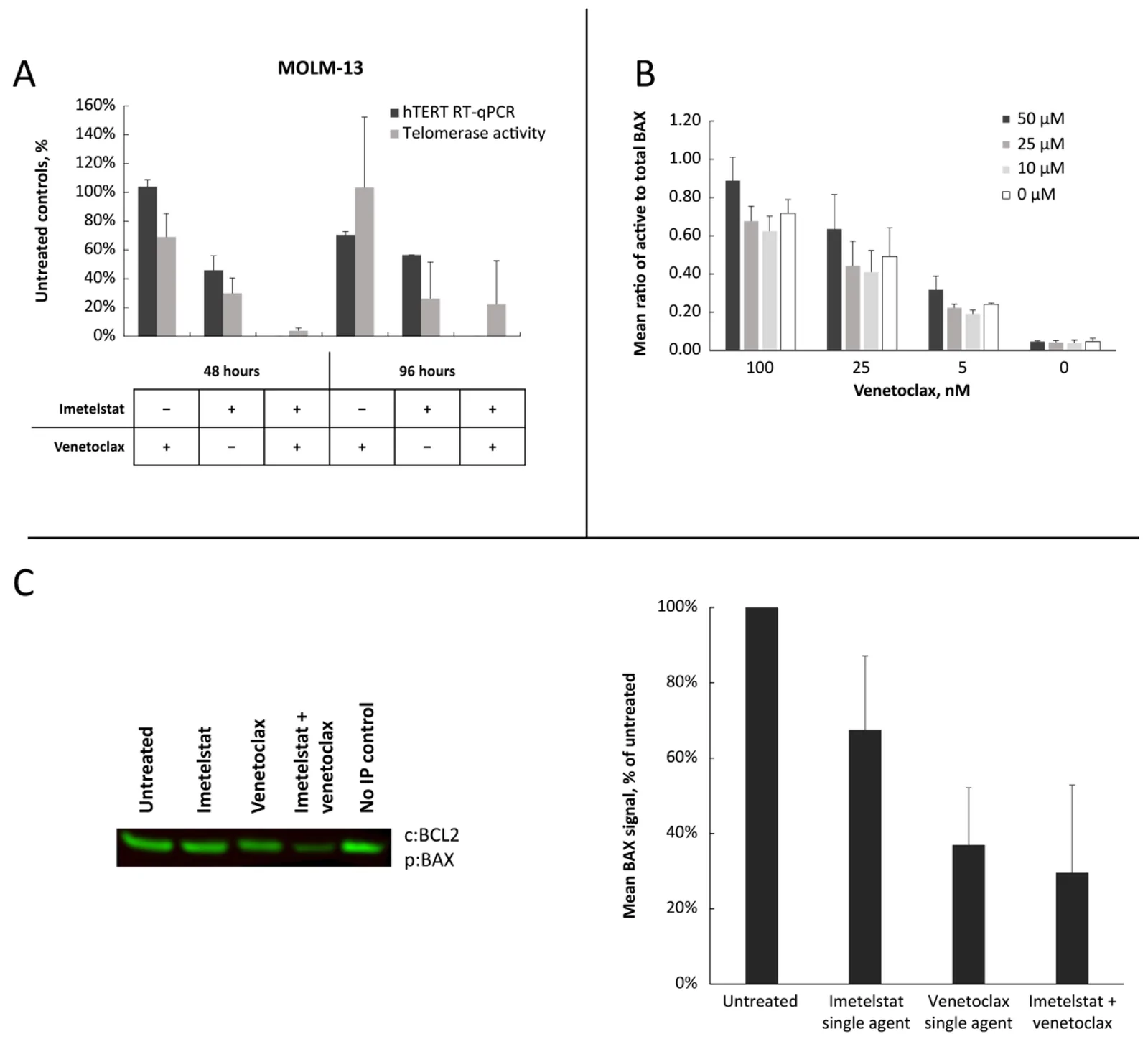
Mechanistic study of synergistic activity produced by combining imetelstat and venetoclax in MOLM-13. (**A**) Reduced expression of hTERT RNA, as measured by RT-qPCR, and telomerase activity, as determined by TRAP-PCR assay. The experiment was performed twice, and results are expressed as mean plus SD. (**B**) Increased in conformationally activated BAX. Cells were treated for 16 h with imetelstat and venetoclax at the concentrations indicated. Total BAX and conformationally active BAX-positive cells were assessed by flow cytometry. The experiment was performed twice, and results are expressed as mean plus SD. (**C**) Decreased interaction of BAX/BCL-2 by 6 h of drug treatments by coimmunoprecipitation and Western blots. Proteins were captured by BCL-2 and probed by BAX. Band signal was reported as percent of the untreated condition. Bars represent means and SDs of three experiments. The graphical figures and the Western blot show representative results from two independent experimental replicates. IP, immunoprecipitation; RT-qPCR, reverse transcription–quantitative polymerase chain reaction; TRAP-PCR, telomeric repeat amplification protocol–polymerase chain reaction.

### 3.7. Imetelstat Caused the Loss of Mitochondrial Membrane Potential, Leading to Apoptosis When Combined with Venetoclax

To investigate if the enhanced synergistic apoptotic effect when imetelstat is combined with venetoclax is through mitochondrial apoptotic signaling, mitochondria membrane potential (Δψm) was tested. Results showed that imetelstat yielded a strong quenching of Δψm-based MitoTracker staining in MOLM-13 cells (Figure 6A, red), and this phenomenon was further confirmed by a flow cytometric assessment of JC-1 accumulation (Figure 6B). Imetelstat treatment caused almost complete diminishment of aggregated JC-1, an indicator of Δψm loss, within 4 h, during which time the cells were not undergoing appreciable apoptosis, suggesting imetelstat may act in part to reduce the threshold that makes the cells more susceptible to apoptosis.

## 4. Discussion

We report for the first time, to our knowledge, the synergistic effect of the combination of the BCL-2 inhibitor venetoclax with the telomerase inhibitor imetelstat at concentrations within the range of clinically relevant exposure [50] in inducing apoptosis in AML cell lines and leukemic blasts from AML patient samples. Three patterns of susceptibility were observed across the cell lines tested. Group 1 (HEL, U937, and NB-4) was not sensitive to imetelstat nor venetoclax as single agents, nor to the combination of imetelstat with venetoclax, and thereby may represent patients with AML who would need an alternative treatment option. Cell lines in Group 2 (MOLM-13, HL-60, and KG-1) showed added activity with the combination of imetelstat plus venetoclax over either single agent alone, and Group 3 (OCI-AML3 and OCI-AML5) had some marginal additive activity. Taken together these results suggest patients could benefit from the combination of imetelstat plus venetoclax; however, further testing in clinical studies is needed. Additionally, the concentrations and dosing schedules used in the present in vitro and in vivo studies do not directly replicate clinical imetelstat exposure. Differences in pharmacokinetics, tissue distribution, and target engagement between experimental models and patients limit direct comparison of effective preclinical and clinical concentrations [51,52]. Nevertheless, these results are consistent with previous research that showed HEL, U937, NB-4, and OCI-AML3 cell lines are generally resistant to venetoclax compared with MOLM-13, HL-60, and KG-1 cell lines [26,53,54]. The enhanced apoptosis by the combination correlated with increases in DNA damage, as measured by γ-H2AX and p53 levels (Figure 2), and loss of mitochondrial membrane potential (Figure 6A,B). Importantly, this combination significantly prolonged survival in AML xenografts in vivo (Figure 4C), suggesting the benefit of co-targeting BCL-2 and telomerase.

Because venetoclax is currently approved in combination with hypomethylating agents in patients with AML [29,30], we performed head-to-head comparisons of venetoclax with imetelstat to venetoclax with either azacytidine or decitabine in MOLM-13 and HL-60 AML cell lines. We observed that the Loewe excess synergy score, an indicator of the synergistic effect to induce apoptosis, was higher with venetoclax plus imetelstat than venetoclax plus azacytidine or decitabine. A limitation of the Loewe additivity model is that it assumes both agents act through similar or convergent mechanisms with parallel dose–response curves, which is not the case for imetelstat and venetoclax, for which drug effects occur on different timescales (imetelstat requires prolonged exposure and venetoclax induces rapid apoptosis) [15,26,55,56]. In this case, the Loewe model may not have captured the true biologic synergy between these two agents [57]. However, the selection of the Loewe model was supported by the fact that although the drugs evaluated target different but interacting pathways, the downstream biological events are not probabilistically independent. In addition, because the Loewe model is built on the idea of dose equivalence, it offers a stable mathematical reference point for combinations where dose–response curves differ, reducing the risk of overestimating synergy in systems where time-dependent effects or nonparallel curves exist [55,58,59,60]. Despite limited statistical rigor and that these assays were conducted in only two cell lines using a flow cytometry viability assay, these results are encouraging. Further evaluations of venetoclax plus imetelstat in other xenograft models of AML, as well as in AML patient-derived xenograft (PDX) models, are recommended to confirm these results.

Resistance to venetoclax plus azacitidine is a major clinical challenge, emerging primarily through mutations in *TP53*, RAS pathway genes, and BAX, as well as through adaptive upregulation of antiapoptotic proteins, including MCL-1 and BCL-xL. Resistance is also driven by metabolic reprogramming [61,62]. It has been reported that imetelstat in combination with azacitidine showed enhanced activity for targeting leukemia stem cells and enhancing survival of PDX models [63]. In addition, adding imetelstat to venetoclax and azacitidine significantly reduced peripheral blood AML donor chimerism of PDX models [64,65]. Beyond the potential mechanisms described in this manuscript, studies in AML models have demonstrated imetelstat induction of lipophagy, promoting the formation of polyunsaturated-fatty-acid-containing phospholipids, which leads to excessive levels of lipid peroxidation and oxidative stress resulting in ferroptosis in AML. The clinical translation of the preclinical synergy of imetelstat with azacitidine ± venetoclax is currently being tested in the Phase 1/1B IMAGINE study (NCT07320235). This actively recruiting study is a two-part trial to evaluate the safety and preliminary efficacy of imetelstat in combination with azacitidine with or without venetoclax in patients with relapsed or refractory AML. The trial consists of a safety run-in phase (part A) employing a 3 + 3 design to monitor dose-limiting toxicities of imetelstat when administered in combination with a fixed dose of azacitidine. Part B will consist of a Phase 1b trial employing a BOIN12 design to determine the optimal biological dose of imetelstat, starting at a lower dose level, in combination with azacitidine and venetoclax. This study will help inform the treatment of older patients with AML who are ineligible for intensive chemotherapy by potentially providing an alternative regimen to venetoclax in combination with azacitidine.

Telomerase activation and BCL-2 deregulation widely occur in cancer cells, and the link between telomerase and BCL-2-mediated antiapoptosis has been reviewed elsewhere [34,66]. A recent genetic assessment study in preclinical AML models demonstrated that, in a subset of AML cells with short telomeres, telomerase inactivation resulted in rapid induction of apoptosis [67]. BCL-2 is implicated in the regulation of telomerase activity and increased hTERT protein levels [35]. Overexpression of hTERT inhibits BCL-2-dependent apoptosis, and silencing of TERT expression sensitizes cells to apoptosis within a brief time, which is shorter than the timing required for a mechanism involving telomere shortening, suggesting extratelomeric effects of hTERT in induction of cell death [36,68]. The relationship of hTERT to the mitochondrial apoptosis pathway has been observed via the inhibition of translocation of BAX to the mitochondria, which decreases mitochondrial membrane potential, and causes the release of cytochrome c [69]. Thus, there was a mechanistic rationale for the synergistic activity of venetoclax plus imetelstat in the induction of apoptosis. To confirm this hypothesis, we first investigated their impact on hTERT RNA expression and telomerase activity and showed that both were decreased by single-agent imetelstat, as expected, and negligibly by single-agent venetoclax; however, substantial reductions in response to the combination were observed (Figure 5A). Because it has previously been reported that the silencing of hTERT leads to growth inhibition that is associated with a decrease in BCL-2 [70], we further tested if imetelstat could modulate the expression of antiapoptotic proteins. Moderate decreases of MCL1, BCL-2, BCL-xL, and BFL1 RNA levels by imetelstat alone or in combination with venetoclax were observed (Supplementary Figure S2A). This may explain in part the synergistic activity of the combination, as it was reported that reduction of MCL-1 and BCL-xL can increase the activity of venetoclax [71], and co-targeting MCL-1 enhances venetoclax activity [72].

Venetoclax is a specific inhibitor of BCL-2 and upon binding releases proapoptotic proteins to induce apoptosis [24]. Venetoclax decreased the BAX/BCL-2 interaction by reducing the amount of BAX bound by BCL-2 (Figure 5C) and strongly promoted the activating conformational shift in BAX (Figure 5B), whereas imetelstat had minimal impact on its own but further augmented the effect of venetoclax in the combination. It was reported that hTERT can translocate from the nucleus to mitochondria following drug treatment or increase during oxidative stress, where it modulates ROS production and blocks BAX translocation to the mitochondria, thus promoting cell survival by inhibiting ROS-induced apoptosis [69,73,74]. Neither imetelstat nor venetoclax had any appreciable impact on the hTERT translocation between nuclei and mitochondria in our studies (Figure 6A, green); however, imetelstat strongly suppressed the staining of mitochondrial-membrane-potential-based dye (Figure 6A, red), indicating mitochondrial dysfunction. This observation was further corroborated by JC-1 assay results (Figure 6B) showing Δψm was almost completely lost within 4 h of imetelstat treatment, during which time cells were not yet appreciably undergoing apoptosis. The profound effect of imetelstat on Δψm loss may indicate that imetelstat potentially lowers the threshold for venetoclax to induce apoptosis via noncanonical extratelomeric effects of hTERT, whose expression is downregulated by imetelstat and further reduced by the combination (Figure 5A). We hypothesize that a potential mechanism for imetelstat synergistically inducing apoptosis with venetoclax is modulated through the reduction of hTERT expression. This then leads to (1) modulation of the dynamic balance of proapoptotic effectors and antiapoptotic proteins toward promoting apoptosis by decreasing the expression of MCL-1 and BCL-xL, thereby sensitizing cells to venetoclax activity, and (2) the triggering of mitochondrial dysfunction by inducing the loss of Δψm to lower the threshold of mitochondrial apoptosis. In addition, the induction of p53 demonstrated in these experiments correlates with previous research exhibiting that decreases in BCL-2 expression in TP53 knock-out AML cell lines results in venetoclax resistance and that activation of p53 can overcome venetoclax resistance [75,76]. Imetelstat has been shown to induce telomere-driven DNA damage [77], which is thought to prime p53-dependent apoptotic signaling and increase levels of p53 protein expression in TERT knockdown cells [67], all of which may contribute to synergistic effects when combined with venetoclax.

Here, for the first time, to the authors’ collective knowledge, we demonstrate a synergistic effect of combining imetelstat with venetoclax in AML. A significant limitation of these results is that comparative statistical analyses were not feasible due to the small sample size and limited times each experiment was performed. Nevertheless, our findings provide a testable hypothesis that warrants clinical investigation of imetelstat in combination with venetoclax in patients with AML. Taken together, the preclinical evidence presented here supports the initiation of the IMAGINE clinical study of imetelstat plus venetoclax, as the outcome of this study will provide insight on this combination as an emerging therapeutic strategy in AML.

## Figures and Tables

**Figure 1:**
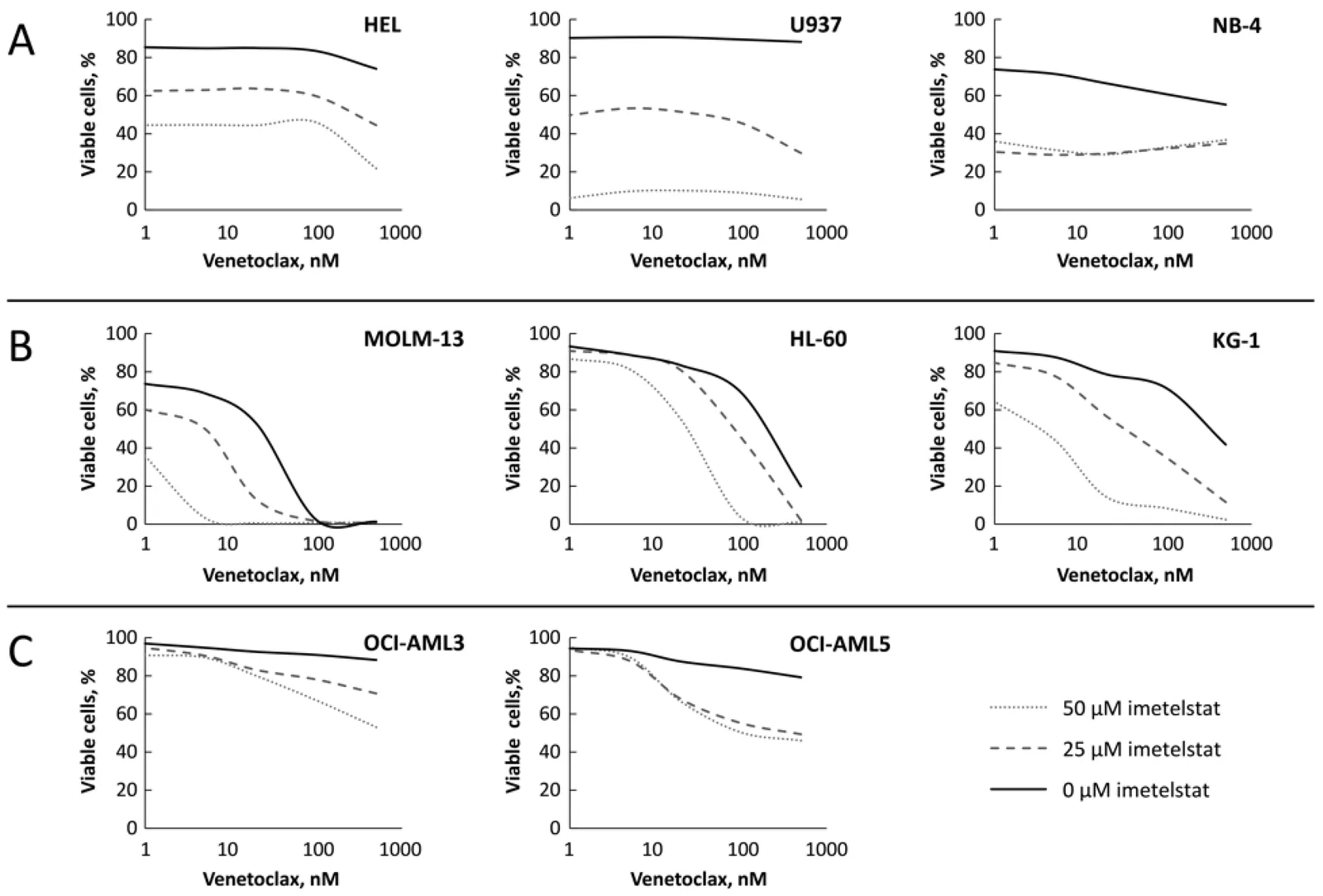
Three distinct patterns of response to imetelstat and venetoclax combination in eight AML cell lines. Each figure shows a representative result from two independent experimental replicates. The vertical axes represent percentage of viable cells. Cells were treated with a range of concentrations of imetelstat and/or venetoclax in vitro for 96 h. The cell viability was assessed by apoptosis assay using flow cytometry. (**A**) HEL, U937, and NB-4. (**B**) MOLM-13, HL-60, and KG-1. (**C**) OCI-AML3 and OCI-AML5. AML, acute myeloid leukemia.

**Figure 2:**
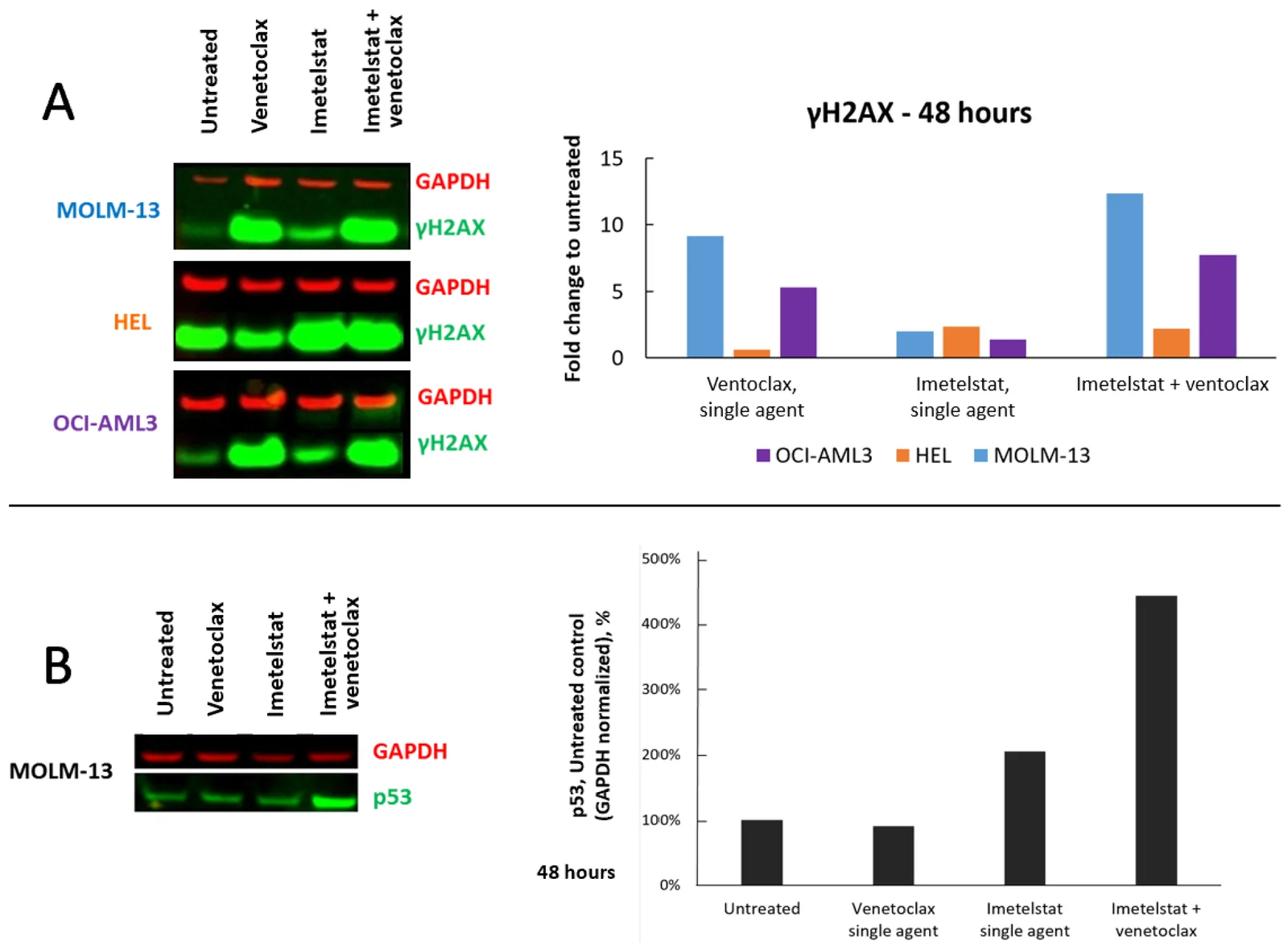
Imetelstat and venetoclax combination increases DNA damage. MOLM-13, HEL, and OCI-AML3 cells were treated with 25 μM imetelstat and 50 nM venetoclax for 48 h. Each figure shows a representative result from two independent experimental replicates. Western blots and quantifiable bar graphs show increased levels of (**A**) γ-H2AX and (**B**) p53 relative to the GAPDH control. GAPDH, glyceraldehyde 3-phosphate dehydrogenase.

**Figure 3:**
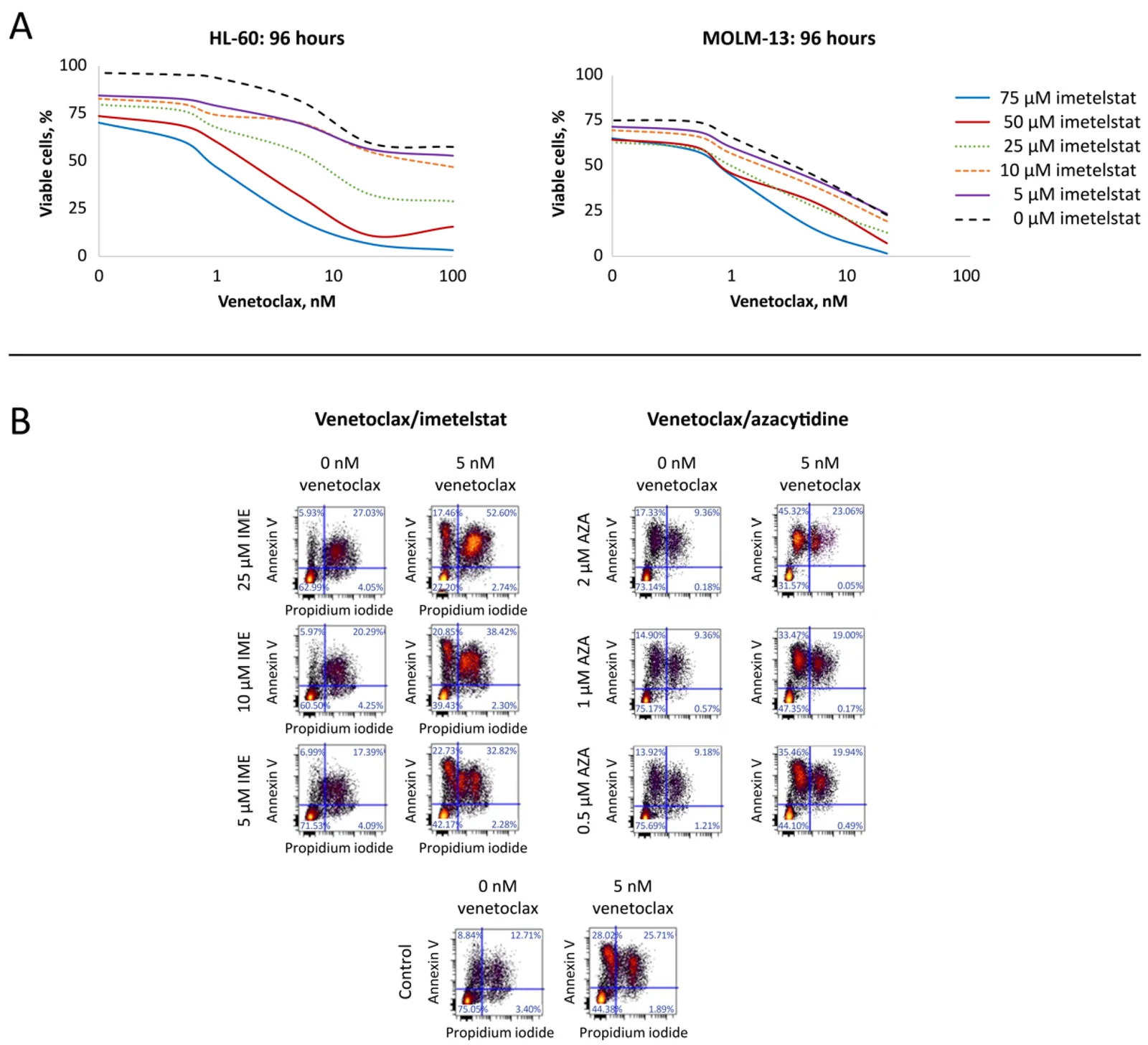
Synergistic evaluation of imetelstat with venetoclax in AML cell lines and comparison to hypomethylating agents with venetoclax in induction of apoptosis. (**A**) imetelstat with venetoclax combination in MOLM-13 (left) and HL-60 (right). (**B**) Representative dot plots from selected doses of imetelstat with venetoclax and azacytidine with venetoclax in the MOLM-13 cell line. Colors reflect event density as displayed by the flow cytometry analysis software, with warmer colors indicating higher cell density. Each figure shows a representative result from two independent experimental replicates. AZA, azacytidine; IME, imetelstat.

**Figure 4:**
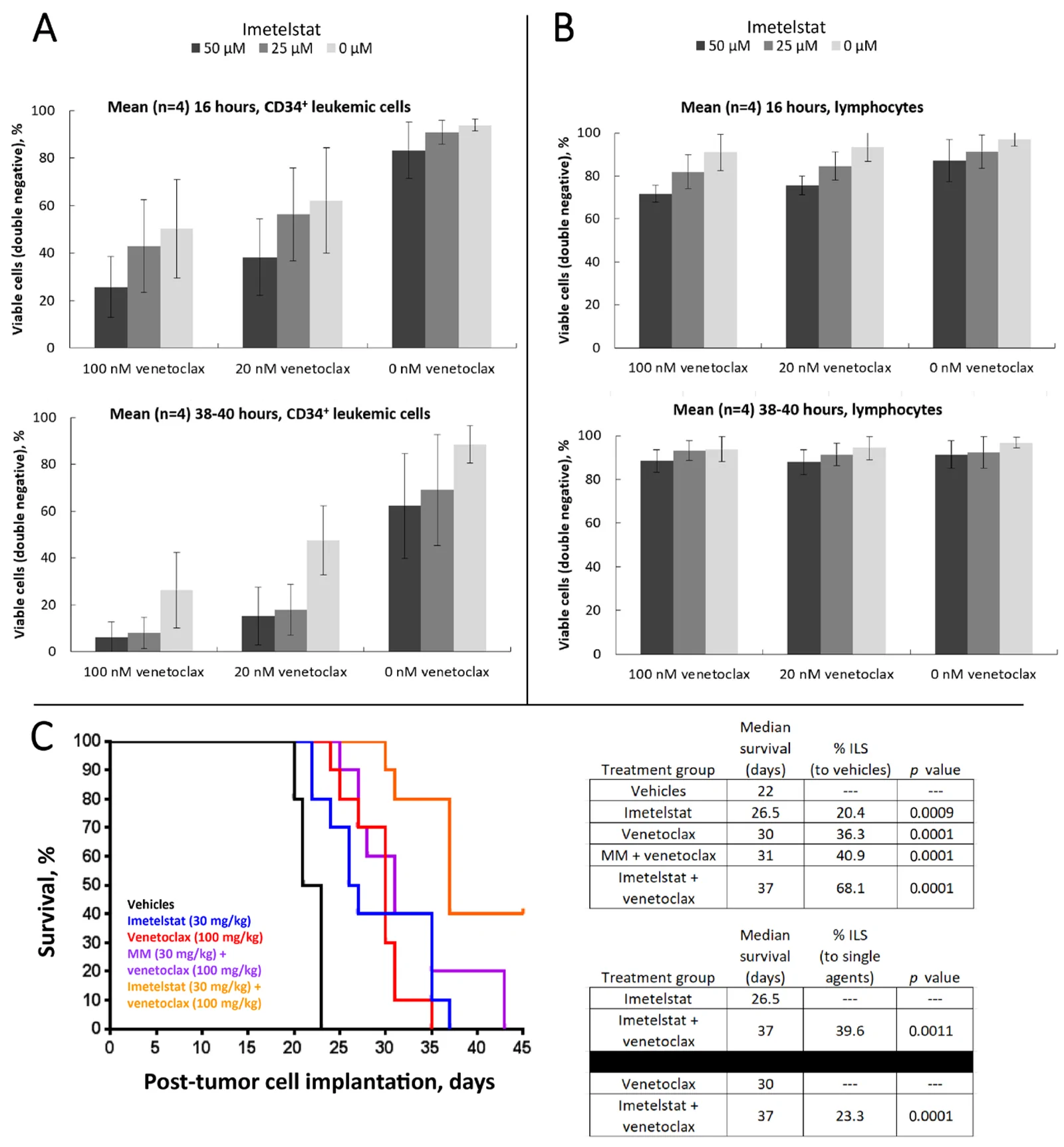
Imetelstat plus venetoclax enhances apoptosis in four AML patient samples ex vivo and prolonged survival of xenograft models in vivo. (**A**) Enhanced apoptosis ex vivo in CD45^dim^/CD34^+^ leukemic cells from primary AML PBMCs (mean of four patients). (**B**) Imetelstat plus venetoclax had no effect on normal lymphocytes (CD45^hi^/side-scatter-low; mean of four patients). (**C**) Survival of MOLM-13 AML xenografts in vivo. Imetelstat (30 mg/kg, three times per week) and venetoclax (100 mg/kg, daily) were given for four weeks. Ten mice per group. Each figure shows a representative result from two independent experimental replicates. Percent ILS was calculated with the *p*-values indicated. Error bars represent standard deviation. ILS, increased life span; PBMC, peripheral blood mononuclear cell.

**Figure 6:**
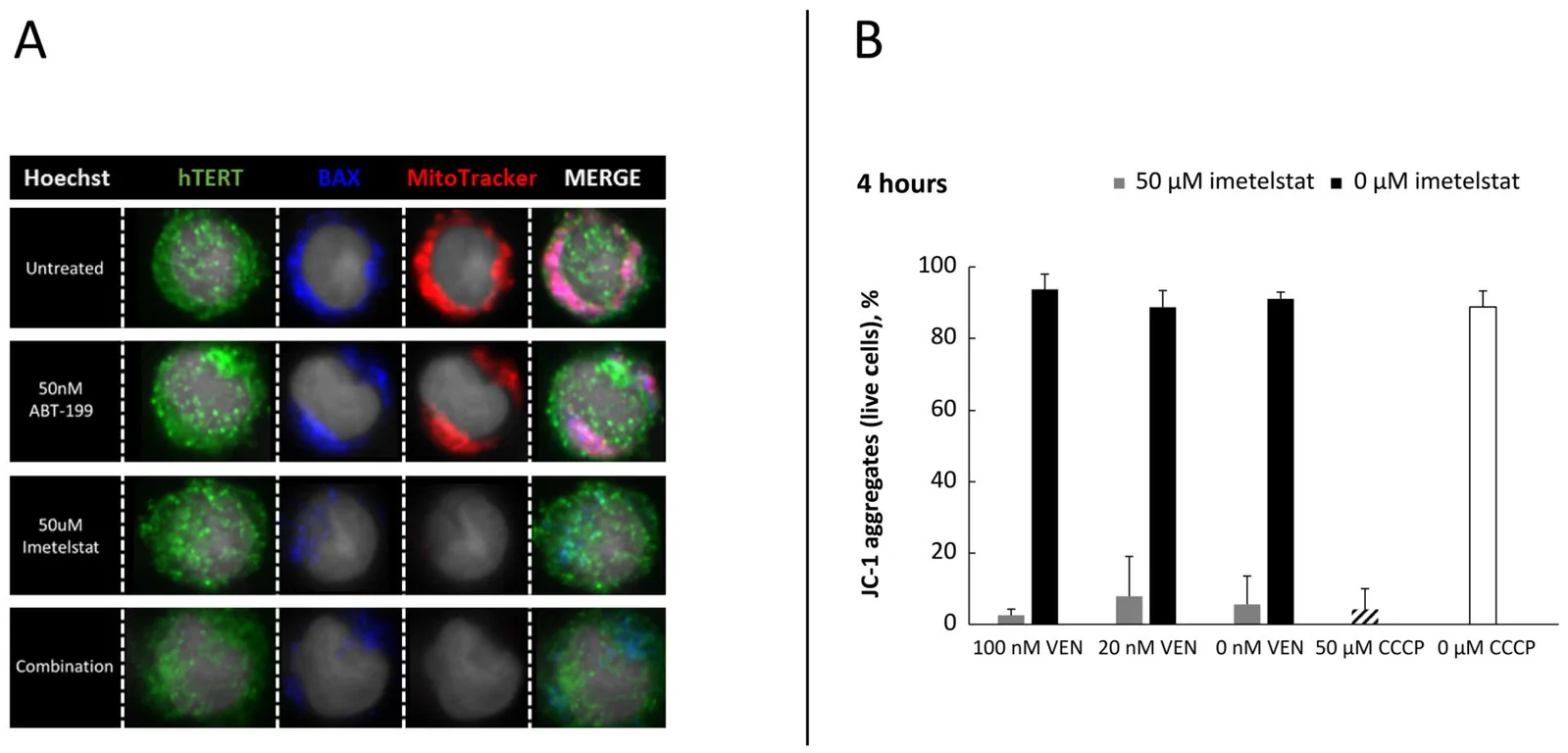
Loss of mitochondrial potential in the MOLM-13 cell line by the imetelstat and venetoclax combination. (**A**) MitoTracker Red staining was dramatically reduced after 2 h of drug treatments. The figure represents results from two independent experimental replicates. (**B**) JC-1 aggregates were also markedly reduced after 4 h of drug treatments. The experiment was performed twice, and results are expressed as mean plus SD. CCCP, carbonyl cyanide m-chlorophenyl hydrazone (control); VEN, venetoclax.

## Data Availability

The datasets presented in this article are not readily available due to technical limitations. Requests to access the datasets should be directed to medinfo@geron.com.

## Supplementary Materials

The following supporting information can be downloaded at: https://www.mdpi.com/article/10.3390/jcm15197399/s1, Supplementary Methods. Figure S1: In vivo efficacy study in AML MOLM-13 xenograft models; Figure S2: Effects of imetelstat in combination with venetoclax on expression of Bcl-2 family members; Table S1: Excess of Loewe’s additivity model of MOLM-13 cells treated with imetelstat and venetoclax for 48 and 96 h in vitro; Table S2: Excess of Loewe’s additivity model of MOLM-13 cells treated with venetoclax and azacytidine or venetoclax and decitabine for 96 h in vitro; Table S3: Excess of Loewe’s additivity model of HL-60 cells treated with venetoclax and imetelstat, venetoclax and azacytidine, or venetoclax and decitabine for 96 h in vitro.

## Abbreviations

AML	acute myeloid leukemia
GAPDH	glyceraldehyde 3-phosphate dehydrogenase
ILS	increased life span
PBMCs	peripheral blood mononuclear cells
PBS	phosphate-buffered saline
PDX	patient-derived xenograft
ROS	reactive oxygen species
RT-qPCR	Reverse transcription–quantitative polymerase chain reaction
SDS-PAGE	sodium dodecyl sulfate–polyacrylamide gel electrophoresis
TRAP-PCR	telomeric repeat amplification protocol–polymerase chain reaction
