# Supplementary material for: Combining the Telomerase Inhibitor Imetelstat with the BCL-2 Inhibitor Venetoclax Enhances Apoptosis and Improves Survival in Acute Myeloid Leukemia Cellular and Animal Models
Source: J Clin Med. 2026 Sep 23;15(19):7399. doi: 10.3390/jcm15197399 (PMC13665851; doi:10.3390/jcm15197399)

Combining the Telomerase Inhibitor Imetelstat With the BCL-2 Inhibitor Venetoclax Enhances Apoptosis and Improves Survival in Acute Myeloid Leukemia Cellular and Animal Models

Supplemental Methods

Below are the patient characteristics of the 4 patients with acute myeloid leukemia (AML) who contributed fresh blood samples that were purchased from a commercial supplier (Discovery Life Sciences, Huntsville, AL, USA).

Patient 1: relapsed disease; female. The patient was diagnosed March 2016. She received 7+3 cytarabine and an anthracycline on 4 March 2016, was diagnosed with refractory disease on 6 March 2016 by bone marrow biopsy, and was also found to have cerebrospinal fluid (CSF)–positive disease, so she received an Ommaya catheter for intrathecal chemotherapy (methotrexate on 21 April 2016; cytarabine on 27 April 2016, 9 May 2016, and 23 May 2016), with subsequently negative CSF cytology. For her refractory disease, she also was enrolled in the UPCC 22415A trial (NCT01729845) and randomized to mitoxantrone, etoposide, and cytarabine (MEC) D1 on 4 May 2016.

Patient 2: de novo disease; female. A 28-year-old female was diagnosed with de novo AML on 5 February 2016. She was induced with 7+3 idarubicin/cytarabine starting 10 February 2016 and achieved complete remission, but her bone marrow at the time showed 0.9% blasts, so she was re-induced with 5+2 idarubicin/cytarabine. She was consolidated with high-dose cytarabine on 25 May 2016. This sample is from when she presented with relapsed disease on 2 September 2016, exhibiting 73% blasts.

Patient 3: de novo disease; female. A 26-year-old female was diagnosed with de novo AML 9 January 2016. She underwent 7+3 induction (cytarabine + an anthracycline) starting 13 January 2016 and achieved complete remission. She was consolidated by 1 cycle of high-dose cytarabine and then had a myeloablative allogeneic stem cell transplant on 28 April 2016. Her bone marrow 100 days after the transplant showed morphologic remission. She presented the week of blood collection with relapsed AML, exhibiting 95% blasts.

Patient 4: presumed acute promyelocytic leukemia (APL); female. This patient was admitted 5 September 2016 with presumed APL. French-American-British designation of AML3 and World Health Organization classification of APL t(15;17)(q22;q12); PML-retinoic acid receptor alpha. She received leukapheresis, and her first dose of all-trans retinoic acid was administered 6 September 2016. Her white blood cell count remained significantly elevated with a presumed high number of promyelocytes or blast equivalents.

Supplemental Table S1. Excess of Loewe's additivity model of MOLM-13 cells treated with imetelstat and venetoclax for 48 and 96 hours in vitro.

Loewe excess; synergy score = 5.12		Imetelstat, µM (48 hours)						
		**0**	**1**	**5**	**10**	**25**	**50**	**75**
Venetoclax, nM	**1000**	-4	-2	-3	3	45	56	56
	**500**	5	10	1	2	44	54	54
	**100**	5	9	12	6	33	51	54
	**20**	0	1	4	1	22	40	51
	**5**	-4	-1	-4	0	7	25	39
	**1**	1	0	-1	-5	-4	6	24
	**0.2**	3	5	7	1	-1	2	14
	**0.04**	13	10	10	9	0	1	8
	**0**		9	7	1	-4	4	2
**Loewe excess; synergy score = 11.33**		**Imetelstat, µM (96 hours)**						
		**0**	**1**	**5**	**10**	**25**	**50**	**75**
Venetoclax, nM	**1000**	-1	5	26	61	71	65	61
	**500**	4	10	24	63	70	65	61
	**100**	0	3	12	38	70	64	61
	**20**	-7	-2	6	22	69	64	59
	**5**	-2	-3	-1	6	19	64	55
	**1**	1	6	0	-1	-1	38	56
	**0.2**	4	4	1	-2	-5	13	53
	**0.04**	9	9	6	0	-5	0	36
	**0**		6	3	-1	-5	-2	5

Supplemental Table S2. Excess of Loewe's additivity model of MOLM-13 cells treated with venetoclax and azacytidine or venetoclax and decitabine for 96 hours in vitro.

**Loewe excess; synergy score = 1.43**		**Azacitidine, µM (96 hours)**					
		**0**	**0.125**	**0.25**	**0.5**	**1**	**2**
**Venetoclax, nM**	**100**	-3	-2	15	21	21	21
	**20**	7	-3	-9	-6	18	23
	**5**	-1	-4	-4	-9	-12	3
	**1**	-3	-2	3	4	0	6
	**0.5**	3	3	4	8	8	8
	**0**		0	-1	-2	-1	1

**Loewe excess; synergy score = 3.46**		**Decitabine, µM (96 hours)**					
		**0**	**0.125**	**0.25**	**0.5**	**1**	**2**
**Venetoclax, nM**	**100**	-2	20	20	20	20	20
	**20**	6	-42	23	23	23	23
	**5**	-3	1	12	27	32	32
	**1**	-3	-4	1	1	29	20
	**0.5**	4	-5	-6	7	18	8
	**0**	0	4	-5	-2	4	3

Supplemental Table S3. Excess of Loewe's additivity model of HL-60 cells treated with venetoclax and imetelstat, venetoclax and azacytidine, or venetoclax and decitabine for 96 hours in vitro.

**Loewe excess; synergy score = 3.06**		**Imetelstat, µM (96 hours)**					
		**0**	**0.125**	**0.25**	**0.5**	**1**	**2**
**Venetoclax, nM**	**100**	0	5	11	29	42	55
	**20**	4	7	8	30	52	56
	**5**	-3	6	5	18	40	52
	**1**	3	6	6	8	13	25
	**0.5**	4	3	1	-1	5	12
	**0**	4	2	-1	-4	0	3
**Loewe excess; synergy score = 1.44**		**Azacitidine, µM (96 hours)**					
		**0**	**0.125**	**0.25**	**0.5**	**1**	**2**
**Venetoclax, nM**	**100**	-3	-4	-13	-6	35	49
	**20**	6	14	13	10	-14	42
	**5**	0	-1	1	-2	-7	-4
	**1**	5	5	5	8	6	6
	**0.5**	3	2	0	3	2	2
	**0**	3	1	0	0	-1	1
**Loewe excess; synergy score = 2.64**		**Decitabine, µM (96 hours)**					
		**0**	**0.125**	**0.25**	**0.5**	**1**	**2**
**Venetoclax, nM**	**100**	-1	6	23	42	45	46
	**20**	4	7	7	2	23	32
	**5**	-2	5	10	16	26	28
	**1**	3	7	5	5	13	17
	**0.5**	2	3	1	1	8	9
	**0**	3	1	-1	-3	3	0

Supplemental Figure S1. In vivo efficacy study in AML MOLM-13 xenograft models. (**A**) Mice were monitored twice weekly for body weight and adverse clinical effects of tumor burden; mice body weights remained relatively consistent for each group for the duration they were monitored. (**B**) Survival curves for in vivo combination study. Prolonged survival in the group treated with the combination of imetelstat and venetoclax was observed, with 40% of mice still being alive at the end of study (>11 weeks after last dose of treatment). Each figure shows a representative result from 2 independent experimental replicates. AML, acute myeloid leukemia, MM, mismatch.

Vehicles: 30 mg/kg mismatch + polyethylene glycol 400/Phosal50/ethanol.

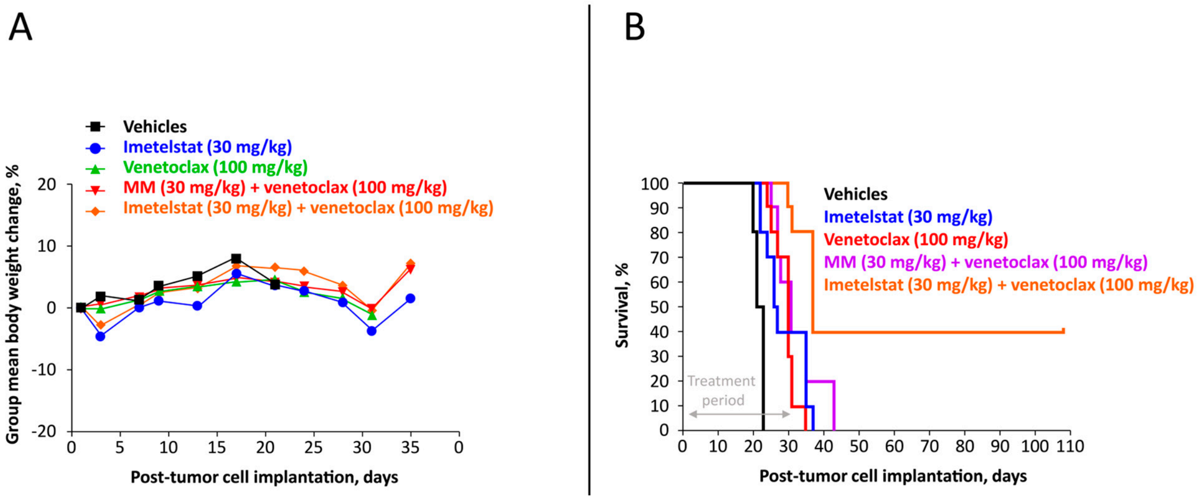

Supplemental Figure S2. Effects of imetelstat in combination with venetoclax on expression of *Bcl-2* family members. (**A**) MCL1, BCL-2, BCL-xL, and BFL1 RNA expression levels by RT-qPCR assay. (**B**) Western blot for protein expression levels of BCL-2, BCL-xL, and BFL1. Each figure shows a representative result from 2 independent experimental replicates. iGAPDH, glyceraldehyde 3-phosphate dehydrogenase; RT-qPCR, reverse transcription-quantitative polymerase chain reaction.

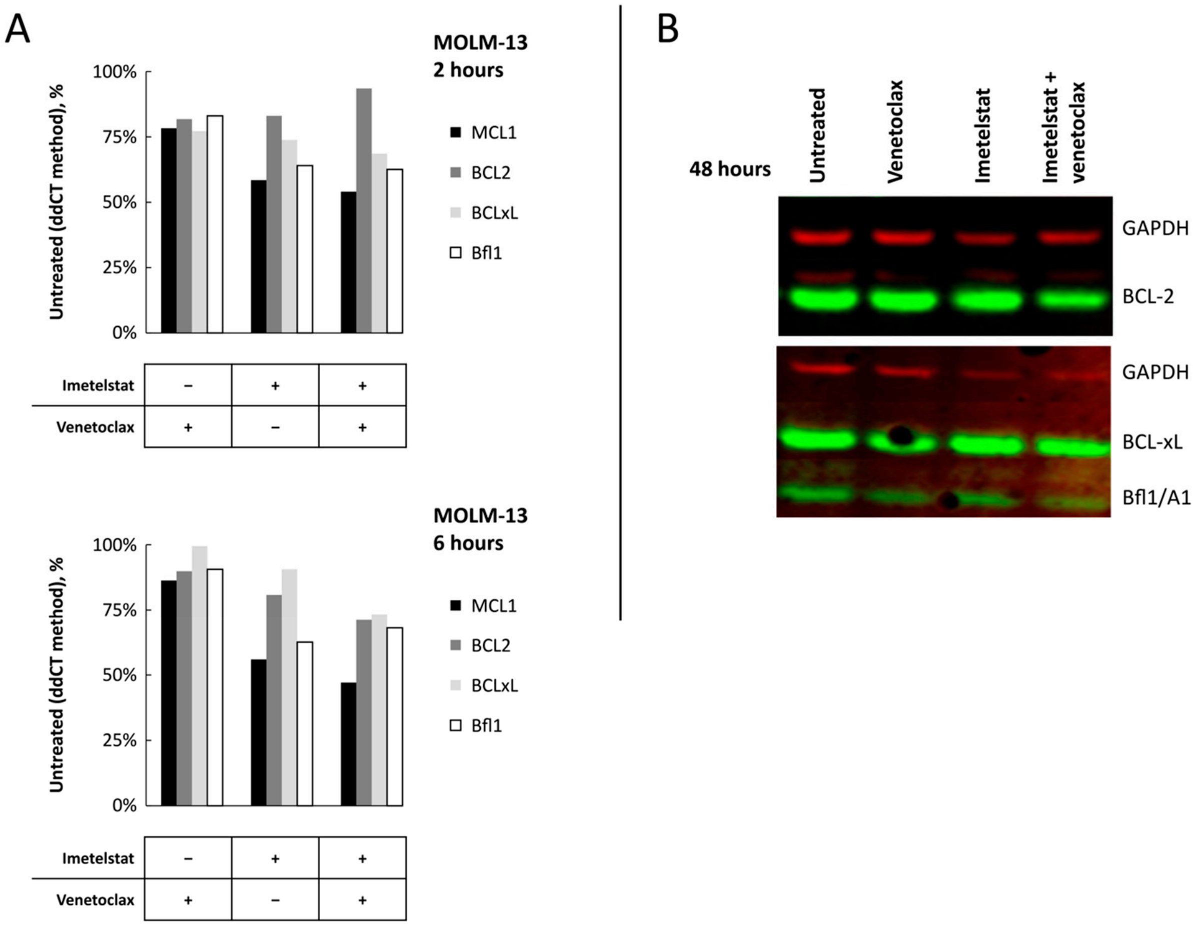

Supplement: Supplementary file 1 [file jcm-15-07399-s001.zip › jcm-4511364-supplementary.pdf]
